# Four Years of Experience Using Problem-Based Learning as a Platform to Develop Student Teaching Skills: A Preliminary Report

**DOI:** 10.15694/mep.2018.0000157.2

**Published:** 2018-08-14

**Authors:** Carla Lupi, Melissa Ward-Peterson, Christian Castro, Ansley Splinter

**Affiliations:** 1 Department of Obstetrics and Gynecology & Office of Medical Education; 2 Department of Obstetrics and Gynecology & Office of Medical Education; 3 Department of Epidemiology; 4 Department of Epidemiology; 5 Madison Teaching and Learning Excellence; 6 Madison Teaching and Learning Excellence; 7 Department of Pediatrics; 8 Department of Pediatrics

**Keywords:** teaching skills, medical students, problem-based learning, feedback, peer-teaching

## Abstract

This article was migrated. The article was marked as recommended.

**Introduction:** A graduating medical student should be competent in both teaching and communication skills. This concept is supported by the Accreditation Council for Graduate Medical Education and the Association of American Medical Colleges. Our study describes how we utilized problem-based learning as a platform for developing student teaching skills and to examine preliminary outcomes.

**Methods:** Since 2013, third-year medical students at Florida International University Herbert Wertheim College of Medicine have participated in a mandatory problem-based learning course in parallel to their clinical rotations. During the course orientation students have been led through interactive sessions on writing learning objectives and methods for effective micro-teaching sessions. During seven subsequent sessions, trained faculty members have assessed and provided narrative comments on students’ “Ability to Teach Peers” using an anchored developmental scale rubric. Data from four academic years were available for analysis. The Wilcoxon signed-rank test was used to test differences between the initial and final sessions.

**Results:** At the initial session, 39.0% (n=147) received ratings of “mastering.” By the final session, 62.6% (n=236) received ratings of “mastering.”

**Conclusion:** Our preliminary work demonstrates that a brief orientation to micro-teaching followed by repeated mandatory practice and feedback within our problem-based learning curriculum may serve to build students’ teaching skills.

## Background

The importance of learning to teach as a key outcome in medical education now has widespread acceptance. The Accreditation Council for Graduate Medical Education (ACGME) competency of practice-based learning and improvement requires that trainees “participate in the education of patients, families, students, residents and other health professionals” ( ACGME, 2009). Indeed, teaching and communication skills underlie three of the Association of American Medical Colleges (AAMC) Entrustable Professional Activites (EPAs), including EPA 6 (provide an oral presentation of a clinical encounter), EPA 8 (give or receive a patient handover to transition care responsibly), and EPA 11 (obtain informed consent for tests and/or procedures) ( AAMC, 2014). The General Medical Council of the United Kingdom states that a graduating student should be able to “work effectively and appropriately as a mentor and teacher for other learners in the multi-professional team” ( General Medical Council, 2018).

In a national survey conducted in 2008, 43 of 99 US medical schools reported offering a formal student-as-teachers programs with the majority occurring as fourth-year electives ( Soriano et al., 2010). Our review of subsequent literature on this topic revealed several additional reports of elective experiences occurring in the fourth year ( Shah et al., 2017; Yeung et al., 2018; Yoon et al., 2017).  While these learning opportunities will support the development of skills among students who choose them, the challenge of promoting competency in all students calls for changes to the mandatory curriculum. With time constraints limiting curricular expansion, educators might identify opportunities in existing curricula to provide students both practice to teach and feedback on their teaching. One such opportunity includes student presentations incorporated within active learning pedagogies such as problem-based learning (PBL).

In a literature review using three databases (PubMed, ERIC, and EMBASE) with the terms “medical education, undergraduate”, “students, medical”, “teaching”, “feedback”, and then same with “problem-based learning,” we found several references to students as near-peer and peer teachers ( Nelson et al., 2013), to electives and modules specifically designed to develop educator skills ( Dandavino et al., 2007), and to programs for developing students as PBL tutors ( Blatt & Greenberg, 2007; Clark et al., 2008). However, we found no literature on the use or impact of feedback to students within problem-based learning curricula to improve their teaching skills.

The objectives of this study are to describe how we utilized PBL as a platform to develop student teaching skills and to examine preliminary outcomes.

## Activity

Since 2013, a third-year mandatory PBL course running parallel to clinical rotations has served as a platform to utilize a rubric and provide feedback on student teaching skills. At our institution, PBL is conducted with groups of 5-7 students. In the morning, they work through an unfolding case in 4-6 segments, making and justifying clinical decisions at each juncture, and identifying learning gaps in the process. For some parts (the history and the laboratory values), they are given only the information they request with justification. The facilitator does not lecture or otherwise provide content. They do not use internet or other resources in the morning, to emphasize use of the group’s collective intelligence. Students then have 2 hours to prepare short individual presentations for the afternoon on their assigned/chosen learning objective(s), during which time they do access the available resources of their choice. The afternoon session consists of the presentations and questions, followed by reflection on the overall session and individual contributions to group learning. Cases are diagnostically challenging, for example a woman in the third trimester of pregnancy with acute appendicitis presenting as pyelonephritis.

During the course student orientation, we conducted interactive sessions on writing learning objectives and structures and guidelines for effective micro-teaching sessions. The latter consisted of a simple structure emphasizing the importance of engaging the audience through initial interaction to activate prior knowledge and ensure connection to the case, use of visual aids (pictures, diagrams, algorithms, etc.) rather than text on PowerPoint to present information, and a closing summary to test understanding and highlight take-away points.

Unable to locate a rubric suitable for the assessment of short, quickly prepared learning sessions, we developed one ourselves. We borrowed the categorical descriptors – emerging, acquiring, mastering – from the PBL rubric then used at the University of New Mexico. The behavioral anchors in the “mastering column” map to the guidelines provided in the orientation session. We used our own knowledge gained through observation of developing faculty to devise the anchors for emerging/developing (2 of us were the primary faculty developers at the time).

Faculty facilitators assessed and provided narrative comments, using this rubric with the domain labeled “ability to teach peers”:

• Emerging (1-2): failed to relate learning issue back to case; rote recital of information from notes and/or PowerPoint; did not engage audience or check for understanding.
• Acquiring (3-4): learning objectives stated or presented in beginning; organized; presented learning issue at appropriate level; related learning issues back to case.
• Mastering (5): engaged audience through the use of frequent questions, visual diagrams, handouts or other activities; checked understanding of audience.

This rubric was discussed in detail at the faculty course orientation, with specific behavioral anchors of student performance at each level.

Data were available for a total of four classes (Classes of 2015-2018). Descriptive statistics included frequency distributions, means, and standard deviations on student performance at the initial and final sessions; the Wilcoxon signed-rank test was used to test differences between the initial and final sessions. The mean of student responses to a Likert-type item included in the post-course survey related to this activity (“The PBL sessions improved my ability to teach effectively”) was also examined from two classes; ratings for this item ranged from 1 (Strongly Disagree) to 5 (Strongly Agree). All analyses were conducted using Stata 14 (College Station, Texas).

This research was deemed exempt from review by the Florida International University Health Sciences Institution Review Board.

## Results

Data were available for both the initial and final sessions for a total of 377 students. Results for these students are shown in Table 1. At the initial session, 39.0% (n=147) received a rating of “mastering”, with a mean score of 4.17 (SD=0.81; range: 1-5). By the final session, 62.6% (n=236) were rated as “mastering”, with a mean score of 4.58 (SD=0.58; range: 3-5). Results of the Wilcoxon signed-rank test indicated a significant difference between the initial and final sessions.

**Table 1.** Four classes of performance data on student ability to teach peers at initial and final sessions in a third-year problem-based learning course

Class	Performance at Initial Session		Performance at Final Session	
	Mean (SD)	Percent Rated As “Mastering”	Mean (SD)	Percent Rated As “Mastering”
Class of 2015 (n=77) ^a^	4.14 (0.94)	44.2%	4.61 (0.61) ^*^	67.5%
Class of 2016 (n=99) ^b^	3.99 (0.79)	26.3%	4.54 (0.56) ^*^	56.6%
Class of 2017 (n=92) ^c^	4.41 (0.76)	56.5%	4.66 (0.54) ^*^	69.6%
Class of 2018 (n=109) ^d^	4.14 (0.72)	32.1%	4.52 (0.62) ^*^	58.7%
Overall (n=377) ^e^	4.17 (0.81)	39.0%	4.58 (0.58) ^*^	62.6%

SD = Standard Deviation

^*^ Statistically significant improvement (p<0.05).

^a^ Total class size: 78; data available for 98.7%.

^b^ Total class size: 114; data available for 86.8%.

^c^ Total class size: 113; data available for 81.4%.

^d^ Total class size: 120; data available for 90.8%.

^e^ Total number of students: 425; data available for 88.7%.

Post-course survey data were available for a total of 186 students from two classes. For the Class of 2016, the mean rating of the statement “The PBL sessions improved my ability to teach effectively” was 4.1 (SD: 0.75; n=83; response rate: 72.8%). The mean rating for the Class of 2018 was 4.0 (SD: 0.96; n=103; response rate: 85.8%). For logistical reasons, data were unavailable for the Classes of 2015 and 2017.

We did not systematically evaluate the quality of narrative feedback, nor investigate its relationship to changes in ratings of student performance.

## Discussion

Our preliminary findings show that initial instruction in basic principles followed by narrative feedback within a problem-based learning course may contribute to improving students’ teaching skills. The lower standard deviation at the final session indicates that ratings of student performance became more standardized over the academic year.

The strengths of our work include the novelty of using PBL as a platform to assess student teaching skills as well as our large sample size. While the one-day version of PBL practiced at our institution is not typical, we can easily see easy transferability of our approach to more traditional versions, and to any version of case-based learning with required prepared peer teaching to address learning gaps.  Another strength is the consistent finding of improvement over the academic year in each of the four classes. An important limitation of this study is our lack of comparison groups that did not engage in the practice or in the instruction on microteaching, and/or receive faculty feedback provided on their teaching. Neither can we isolate effects of the PBL course from the clerkships where students may receive feedback on didactic presentations. However, the PBL sessions serve as the most standardized and frequent didactic teaching practice our students receive in their third year. Additionally, student responses in the post-course survey support their perception that the PBL sessions played a key role in improving their teaching skills. Data for this question from all four of the classes would have strengthened this support.  Finally, we have not yet been able to systematically explore possible validity evidence for our rating scale; we plan to do so in future studies.

## Conclusion

Our preliminary work demonstrates that a brief orientation to micro-teaching followed by repeated mandatory practice and feedback within our problem-based learning curriculum may build students’ teaching skills, as judged by faculty ratings as well as student perception. Future research should explore the content of narrative feedback from faculty with respect to effectiveness and developmental appropriateness. Our work has also uncovered the need to devise rating systems for use in  PBL and other case-based settings with validity evidence to judge the quality and impact of student teaching and provide rigorous outcome assessment for future work in this area.

## Take Home Messages

• To our knowledge, this is the first report on the use of mandatory PBL or case-based curricula to develop medical student teaching skills.
• Mandatory PBL and other case-based learning platforms requiring peer teaching may serve as an efficient platform for the development and improvement of medical student teaching skills.
• Medical students perceive that a problem-based learning course offers the opportunity to develop teaching skills.
• Orientations to micro-teaching can be easily incorporated into a PBL course orientation; quantitative and qualitative ratings of student micro-teaching performance can be easily incorporated into PBL rubrics.

## Notes On Contributors

**Dr. Carla S. Lupi** is the Associate Dean for Faculty and Professor of Obstetrics and Gynecology at Florida International University Herbert Wertheim College of Medicine, where she also serves on the team working with the AAMC Pilot for the Core Entrustable Professional Activities for Entering Residency.

**Dr. Melissa Ward-Peterson** is a Postdoctoral Associate in Department of Epidemiology, Robert Stempel College of Public Health and Social Work, Florida International University. She is currently supported by the National Institute of Minority Health and Health Disparities grant (1U54MD012393-01) for FIU-RCMI.

**Dr. Christian Castro** is Assistant Director for Teaching and Learning, Collaborative for Advancing Teaching and Learning, University of Wisconsin-Madison.

**Dr. Ansley Splinter** is an Assistant Professor of Pediatrics at Nationwide Children’s Hospital and The Ohio State University.
