# Supplementary material for: Four Years of Experience Using Problem-Based Learning as a Platform to Develop Student Teaching Skills: A Preliminary Report
Source: MedEdPublish (2016). 2018 Aug 14;7:157. Originally published 2018 Jul 26. [Version 2] doi: 10.15694/mep.2018.0000157.2 (PMC10701815; doi:10.15694/mep.2018.0000157.2)
Supplement: Peer review history

# Reviewer report: Reviewer response for version 2

**DOI:** 10.21956/mep.20223.r31321

**Published:** 2019-03-14

**Authors:** Richard Hays

**Affiliations:** 1 James Cook University

This review has been migrated. The reviewer awarded 3 stars out of 5 An interesting feature of this paper is that the authors are making explicit something that has probably been implicit: students behaviour during PBL group discussions can be used for purposes other than assisting with integration and application of knowledge. Leadership is often mentioned, but the benefits are likely to relate to the focus of the observation and feedback. Developing education skills is a suitable focus, although care must be taken not to to detract from the main aim of the PBL discussions. Care must also be taken not to over-interpret any effect, as small group facilitation is only one of many skills that medical educators need.

Reviewer Expertise:

NA

No decision status is available

# Reviewer report: Reviewer response for version 2

**DOI:** 10.21956/mep.20223.r31320

**Published:** 2018-08-17

**Authors:** Trevor Gibbs

**Affiliations:** 1 AMEE

This review has been migrated. The reviewer awarded 4 stars out of 5 Thank you for revising the paper and clarifying the methods ( your hybrid model of PBL) better, it certainly brings it into a clearer perspective. If an example of the case could have been provided it would have made it clearer if this was a case-based (CBL) study or a hybrid PBL. I still have worries about the methodology- sorry. I am not sure that a "true" PBL activity can be done in such a short period of time, particularly the discussive element and the possibility of addressing all three learning domains. However, and despite my reservations the authors have show an improvement in their students' teaching ability. Whether this was a result of the PBL, a result of the repetitive action accompanied by feedback or just increased confidence, I do not think that this paper can confidently confirm; there would have to be a comparative cohort to show this. However I would still like to congratulate the authors again on their attempt to create a way in which students improve their teaching skills, whilst still developing their clinical skills; both I feel being so closely tied.

Reviewer Expertise:

NA

No decision status is available

# Reviewer report: Reviewer response for version 1

**DOI:** 10.21956/mep.18982.r27199

**Published:** 2018-08-13

**Authors:** Trevor Gibbs

**Affiliations:** 1 AMEE

This review has been migrated. The reviewer awarded 2 stars out of 5 An interesting although a rather confusing paper.I have not seen a "PBL" course that has been shortened to one day and like my colleagues I would not call what the authors describe here as a PBL session. It would have been helpful to see some of the various scenarios used to differentiate them from a case-based learning approachThat said, I also worry that the authors directly attribute the improvement in the students teaching to the PBL, given that they already had attended an orientation session on teaching and learning, had seven "goes" at teaching or presenting ( as the authors state", and had received some form of feedback through that seven opportunities time. I feel that there needed to be at least a comparative group who had not received those extra inputs to see if it was the PBL that brought about improvement.How we improve "teaching" within our graduates is very important since in everyday of our professional lives we teach, to patients, co-workers, peers, near-peers and such like. How we develop that is often found in our communication skills training, but we need to look at other methods. I congratulate the authors in attempting to combine teaching development with curricula learning, although in this case I remain to be convinced.

Reviewer Expertise:

NA

No decision status is available

# Reviewer report: Reviewer response for version 1

**DOI:** 10.21956/mep.18982.r27201

**Published:** 2018-08-05

**Authors:** Sateesh Babu Arja

**Affiliations:** 1 Avalon University School of Medicine

This review has been migrated. The reviewer awarded 3 stars out of 5 I did read this paper with much interest. The way authors described the way of conducting PBL is away from the normal process of conducting PBL. The normal process of PBL as described includes seven steps; identifying and clarifying the keywords, define the problem, analyze the problem, brainstorm the solutions, develop learning objectives, self study, and then sharing the information and synthesis and evaluation. May be it was implemented peer teaching at the level of sharing the information. It seems interesting. But I didn’t see that time was given for self-study or gathering the information as authors described their PBL sessions as one day sessions. The writing style is simple and easy understandable for academic faculty members.

Reviewer Expertise:

NA

No decision status is available

# Reviewer report: Reviewer response for version 1

**DOI:** 10.21956/mep.18982.r27200

**Published:** 2018-08-03

**Authors:** P Ravi Shankar

**Affiliations:** 1 American International Medical University

This review has been migrated. The reviewer awarded 4 stars out of 5 This is an interesting and well-written manuscript. It is important for medical students to be able to teach peers, colleagues, patients and the public as mentioned in the article. Third year medical students used PBL sessions to teach other members of the group and their performance were assessed by faculty members. All students were assessed and the results show a significant improvement in student performance as teachers. I assume that the faculty comments were based on the student presentations during the PBL. The authors have mentioned the limitations of their study and the take home points. I would be interested in greater information about the rubric used for assessing the student performance. The article will be of broad interest to medical educators.

Reviewer Expertise:

NA

No decision status is available

# Reviewer report: Reviewer response for version 1

**DOI:** 10.21956/mep.18982.r27198

**Published:** 2018-07-28

**Authors:** Neslihan Onder-Ozdemir

**Affiliations:** 1 The University of Sheffield

This review has been migrated. The reviewer awarded 3 stars out of 5 Thank you very much for your manuscript that focused on Problem-Based Learning (PBL) as an ‘education strategy’ in medical education. The gap regarding PBL in medical education has been reported very well: there is a paucity of study on “the use or impact of feedback to students within problem-based learning curricula to improve their teaching skills”.Below are my comments to improve the content.Methods section in the Abstract:-In the Methods section in the abstract, the number of the students who participated in the PBL should be stated given that abstract is crucial, and PBL is usually performed with a small group of learners but also a larger group.Methods section in the manuscript:- Some information is needed whether Developmental Scale Rubric reported in this study has been developed by the researchers themselves. If so, some references could be provided how the three criteria have been chosen: Emerging, Acquiring, Mastering. This rubric could be beneficial for medical teachers if they want to use. -Given that PBL is based on clinical problem, to contextualise this research and to show how this rubric can be used, a problem scenario from the research setting can be reported. - In the Discussion part, authors reported as follows: “Our preliminary findings show that initial instruction in basic principles followed by narrative feedback within a problem-based learning course may contribute to improving students’ teaching skills.” In the Abstract: “rained faculty members have assessed and provided narrative comments on students’ “Ability to Teach Peers”. Please provide some data regarding faculty members’ assessment given that this research highlighted the significance of the use or impact of feedback to students within PBL.-There is a need to report about the resource materials used to use PBL (handouts or instructions during the teaching?)All in all, specific examples in the Methods section could make the PBL more concrete for the medical teachers.

Reviewer Expertise:

NA

No decision status is available

# Reviewer report: Reviewer response for version 1

**DOI:** 10.21956/mep.18982.r27197

**Published:** 2018-07-27

**Authors:** Michelle McLean

**Affiliations:** 1 Bond University

This review has been migrated. The reviewer awarded 2 stars out of 5 Thank you for asking me to review this short submission. As a someone who believes that we should all be on the same page when we talk or write about PBL or TBL (highly developed educational approaches underpinned by a series or steps or processes), there is not enough in this article to convince me that the method being described is PBL, i.e. Case Opening with five steps, a period (often a week) of self-study (Step 6), then returning for the Case Closing or Wrap-up (final one or two steps, with Step 7 being elaboration and application and Step 8 being reflection). PBL was designed as a whole curriculum approach so the fact that there were only seven one-day sessions in the academic year suggests that what is being described as PBL may not in fact be PBL in terms of the intended approach? The exercise of formally getting students to 'teach' is, however, well described and is something that could be introduced anywhere in the curriculum where the focus is on patient cases. The template and rubric could be shared with the medical education community. I could, however, see how students could contribute to peer learning during Step 6 by providing the 'expertise' in a hybrid PBL program, i.e. the supplementary sessions to assist learners to meet the learning issues they identified in Steps 1-5 as they worked through a patient presentation. How this currently works in one day (as described) is a difficult to imagine.

Reviewer Expertise:

NA

No decision status is available
